# Supplementary material for: Adaptive Divergence under Gene Flow along an Environmental Gradient in Two Coexisting Stickleback Species
Source: Genes (Basel). 2021 Mar 18;12(3):435. doi: 10.3390/genes12030435 (PMC8003309; doi:10.3390/genes12030435)
Supplement: Supplementary file 1 [file genes-12-00435-s001.pdf]

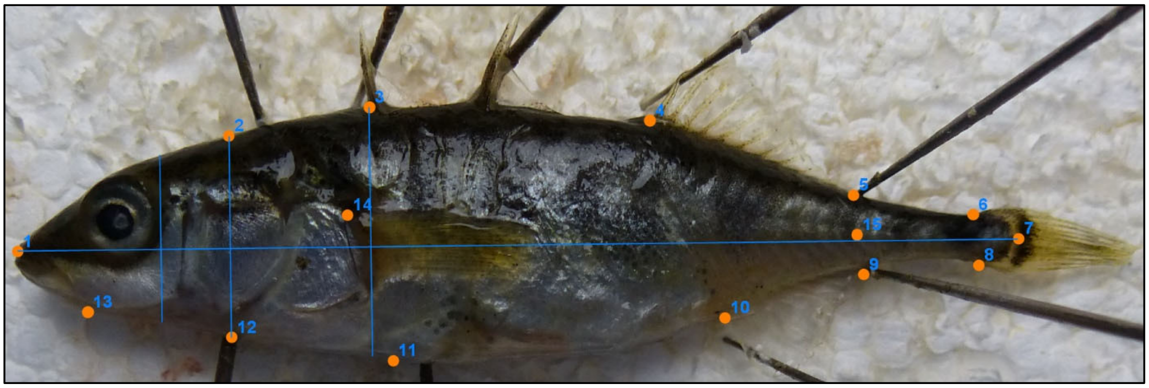

Figure S1. Example of landmarks used for geometric morphometric analysis of body shape, here visualised on the body of a three-spined stickleback individual. For all nine-spined stickleback the same homologous landmarks were used. These 15 landmarks represent a subset of the 19 landmarks that were specifically used for three-spined stickleback in Sharpe *et al*. 2008 ([35] in main paper).

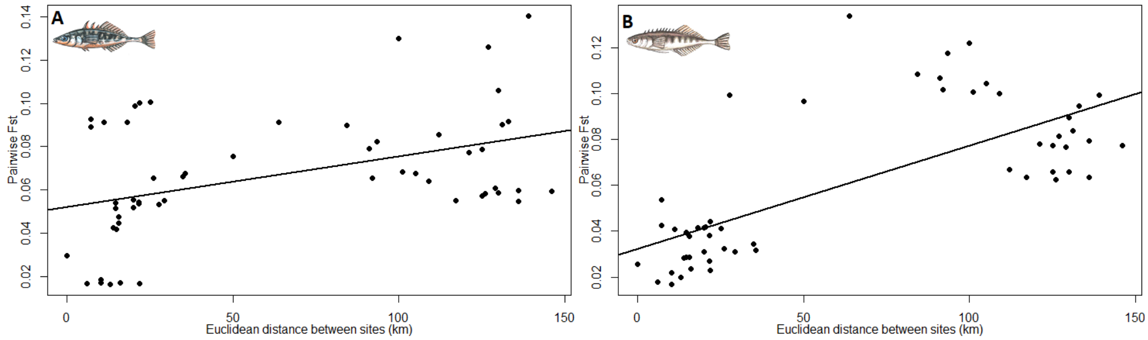

Figure S2. The relationship between Euclidean distances between sites and pairwise F_{ST} in (A) three-spined stickleback (r = 0.42, P = 0.0487) and (B) nine-spined stickleback (r = 0.72, P = 0.0055).

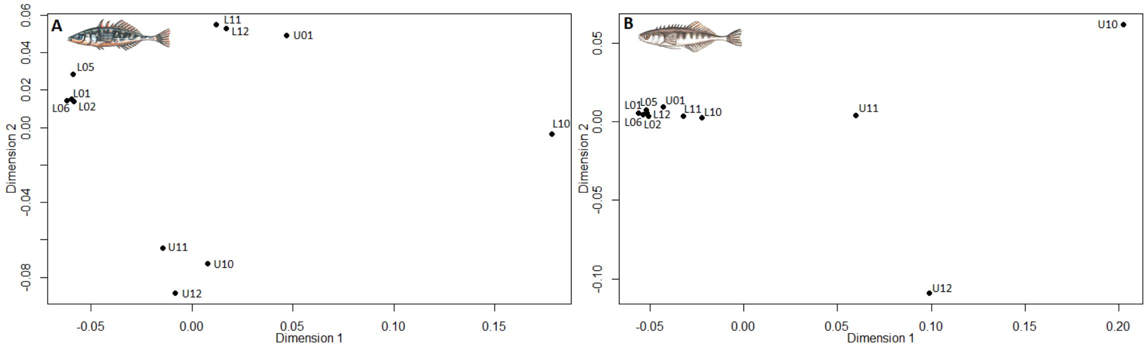

Figure S3. Two-dimensional classical multidimensional scaling of pairwise F_{ST} values in (A) three-spined stickleback and (B) nine-spined stickleback.

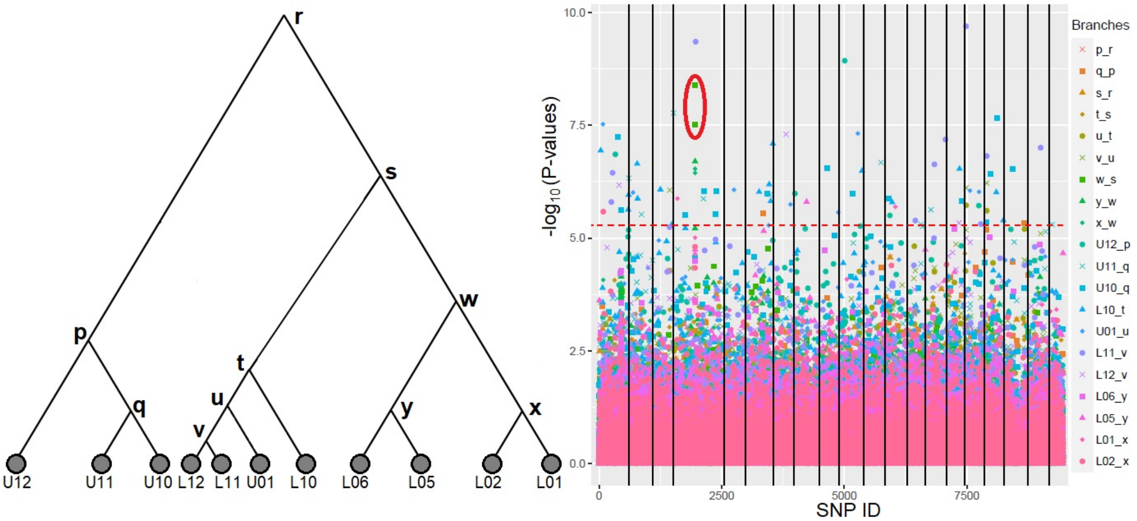

Figure S4. GRoSS v1.0 outlier analysis in three-spined stickleback SNPs along the full genealogy of the eleven populations resulting in a total of 20 outlier tests for each SNP. The horizontal black lines mark linkage groups in chronological order. All SNPs above the red-dashed line represent outliers. The red circle marks two outlier SNPs identified along the w-s branch on LG IV, representing the root of the split between brackish and freshwater sites.

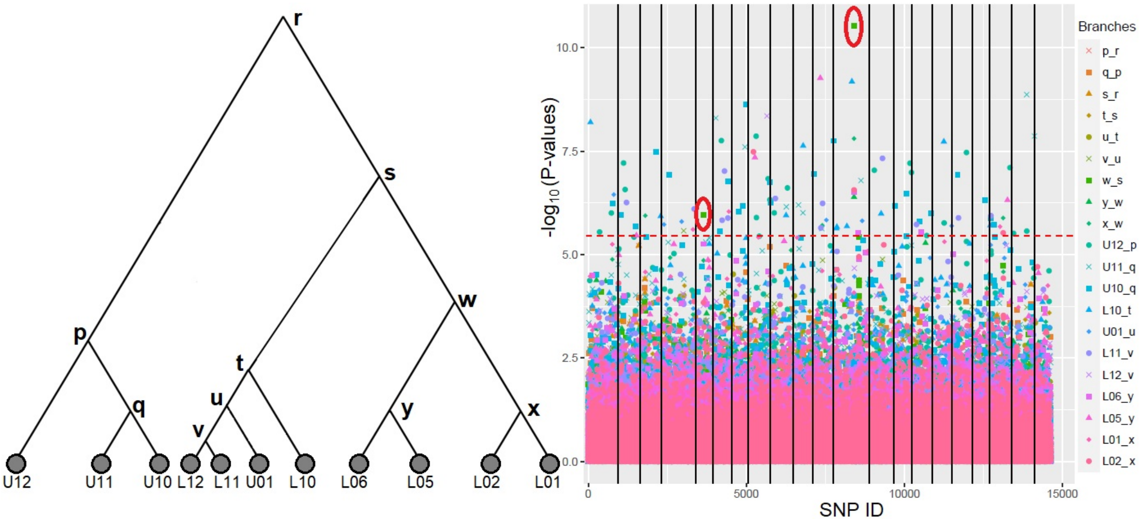

Figure S5. GRoSS v1.0 outlier analysis in nine-spined stickleback SNPs along the full genealogy of the eleven populations resulting in a total of 20 outlier tests for each SNP. The horizontal black lines mark linkage groups in chronological order. All SNPs above the red-dashed line represent outliers. The red circles mark two outlier SNPs identified along the w-s branch on LG V and LG XII, representing the root of the split between brackish and freshwater sites.

Table S1. Pairwise F_{ST} values in three-spined and nine-spined stickleback for eleven sites, with the values for three-spined stickleback above the diagonal and the values for nine-spined stickleback below the diagonal.

	**L01**	**L02**	**L05**	**L06**	**L10**	**L11**	**L12**	**U01**	**U10**	**U11**	**U12**
**L01**		0.017	0.017	0.016	0.100	0.052	0.056	0.066	0.068	0.059	0.060
**L02**	0.018		0.018	0.017	0.101	0.054	0.054	0.068	0.069	0.058	0.061
**L05**	0.024	0.022		0.017	0.091	0.045	0.048	0.055	0.064	0.055	0.060
**L06**	0.020	0.017	0.023		0.099	0.052	0.054	0.065	0.066	0.055	0.057
**L10**	0.044	0.041	0.041	0.042		0.093	0.089	0.091	0.130	0.126	0.140
**L11**	0.041	0.038	0.038	0.040	0.054		0.030	0.042	0.082	0.077	0.092
**L12**	0.031	0.027	0.029	0.029	0.042	0.026		0.043	0.079	0.079	0.090
**U01**	0.035	0.032	0.031	0.032	0.042	0.039	0.028		0.090	0.086	0.106
**U10**	0.105	0.101	0.100	0.102	0.122	0.118	0.107	0.109		0.053	0.091
**U11**	0.066	0.062	0.064	0.064	0.081	0.078	0.066	0.067	0.100		0.076
**U12**	0.080	0.077	0.077	0.077	0.100	0.095	0.084	0.090	0.134	0.097	

Table S2. The P_{ST} values and the corresponding 95% confidence intervals (CI) of 17 morphological traits in three-spined stickleback ([3s]) and nine-spined stickleback ([9s]) on the full geographical scale LBW-LFW-UFW.

	**P_{ST} [3s]**	**P_{ST} 95% CI [3s]**	**P_{ST} [9s]**	**P_{ST} 95% CI [9s]**
**SL**	**0.4896**	**0.4264 - 0.5482**	**0.268**	**0.1941 - 0.3412**
**Plates**	**0.3359**	**0.2673 - 0.4018**	0.07812	0.02857 - 0.1367
**PS**	**0.3934**	**0.3256 - 0.4575**	0.09732	0.04455 - 0.1573
**PP**	0.1438	0.08767 - 0.2045	0.0563	0.01504 - 0.1076
**DS**	**0.124**	**0.07181 - 0.181**	**0.2305**	**0.1611 - 0.3016**
**Eye**	0.2622	0.195 - 0.3289	0.079	0.03365 - 0.1324
**DF**	0.03045	0.001812 - 0.07094	0.01284	0.0006293 - 0.04452
**AF**	0.07287	0.02886 - 0.1246	0.01312	0.0005333 -0.04575
**Tail**	**0.1421**	**0.08579 - 0.2027**	0.06391	0.02096 - 0.1159
**BD**	0.2895	0.2144 - 0.3632	0.06874	0.02107 - 0.126
**NLGR**	0.1065	0.05428 - 0.165	0.03845	0.002167 - 0.08992
**GA**	0.04582	0.01109 - 0.09069	0.04088	0.008021 - 0.08922
**LGR2**	**0.2715**	**0.2044 - 0.3381**	0.08127	0.03574 - 0.1362
**LGR3**	**0.2347**	**0.1695 - 0.3011**	0.09468	0.04467 - 0.1534
**LGR4**	**0.2516**	**0.1853 - 0.3186**	**0.1665**	**0.1015 - 0.2357**
**RW1**	0.0707	0.03533 - 0.1136	0.07122	0.03395 - 0.1172
**RW2**	0.15	0.09864 - 0.2057	0.08888	0.04643 - 0.1391

Table S3. The P_{ST} values and the corresponding 95% confidence intervals (CI) of 17 morphological traits in three-spined stickleback ([3s]) and nine-spined stickleback ([9s]) on the LBW-LFW scale.

	P_{ST} [3s]	P_{ST} 95% CI [3s]	P_{ST} [9s]	P_{ST} 95% CI [9s]
**SL**	**0.4833**	**0.4041 - 0.5549**	**0.3111**	**0.2188 - 0.4008**
**Plates**	**0.1837**	**0.1103 - 0.2602**	0.0783	0.01793 - 0.1508
**PS**	**0.2322**	**0.1534 - 0.3115**	0.08885	0.02755 - 0.1626
**PP**	0.1209	0.05832 - 0.191	0.01105	0.0004783 - 0.04456
**DS**	0.1209	0.0579 - 0.1912	0.1035	0.03733 - 0.1814
**Eye**	**0.2926**	**0.2092 - 0.3736**	0.02199	0.002171 - 0.06454
**DF**	0.02472	0.0007702 - 0.07087	0.01377	0.0004991 - 0.05275
**AF**	0.04779	0.005422 - 0.1039	0.01509	0.0004617 - 0.05702
**Tail**	0.1462	0.07887 - 0.219	0.01243	0.0005503 - 0.04798
**BD**	**0.3418**	**0.2485 - 0.4306**	0.09314	0.0284 - 0.1699
**NLGR**	0.04627	0.002088 - 0.1076	0.02486	0.0004155 - 0.08547
**GA**	0.06528	0.01706 -0.1259	0.05454	0.00903 - 0.1221
**LGR2**	**0.3024**	**0.2198 - 0.3831**	0.05764	0.01522 - 0.1189
**LGR3**	**0.2626**	**0.1823 - 0.343**	0.0707	0.02052 - 0.1382
**LGR4**	**0.2811**	**0.1994 - 0.3613**	**0.1473**	**0.07069 - 0.2321**
**RW1**	0.05469	0.01961 - 0.1015	0.0598	0.02046 - 0.1131
**RW2**	0.1138	0.05854 - 0.1778	0.08022	0.03219 - 0.1409

Table S4. The P_{ST} values and the corresponding 95% confidence intervals (CI) of 17 morphological traits in three-spined stickleback ([3s]) and nine-spined stickleback ([9s]) on the LBW-UFW scale.

	P_{ST} [3s]	P_{ST} 95% CI [3s]	P_{ST} [9s]	P_{ST} 95% CI [9s]
**SL**	**0.4672**	**0.3852 - 0.5417**	0.1752	0.09392 - 0.2618
**Plates**	**0.4557**	**0.3712 - 0.5324**	0.00917	0.0002633 - 0.04082
**PS**	**0.4752**	**0.3921 - 0.5506**	0.04984	0.006588 - 0.1116
**PP**	**0.1709**	**0.09565 - 0.2511**	0.08504	0.02219 - 0.1617
**DS**	0.1359	0.06892 - 0.2101	**0.2309**	**0.1414 - 0.3216**
**Eye**	0.09692	0.03959 - 0.1644	0.1267	0.05738 - 0.2053
**DF**	0.02626	0.0004488 - 0.07839	0.03328	0.001372 - 0.09297
**AF**	0.09286	0.03211 - 0.1648	0.02972	0.0009508 - 0.08837
**Tail**	0.1033	0.03977 - 0.1771	0.08769	0.02684 - 0.1612
**BD**	0.06288	0.01007 - 0.1323	0.02837	0.001176 - 0.08305
**NLGR**	**0.1482**	**0.07141 - 0.2322**	0.04871	0.002025 - 0.1183
**GA**	0.01746	0.001342 - 0.05522	0.02781	0.003191 - 0.07454
**LGR2**	**0.2575**	**0.1737 - 0.3405**	0.1253	0.05544 - 0.2068
**LGR3**	**0.2324**	**0.1501 - 0.3155**	0.1205	0.05188 - 0.1999
**LGR4**	**0.2662**	**0.1817 - 0.3501**	**0.2251**	**0.1366 - 0.3159**
**RW1**	0.1004	0.04627 - 0.1647	0.08101	0.03254 - 0.1416
**RW2**	0.1113	0.05668 - 0.1742	0.08829	0.03677 - 0.1518

Table S5. The P_{ST} values and the corresponding 95% confidence intervals (CI) of 17 morphological traits in three-spined stickleback ([3s]) and nine-spined stickleback ([9s]) on the LFW-UFW scale.

	P_{ST} [3s]	P_{ST} 95% CI [3s]	P_{ST} [9s]	P_{ST} 95% CI [9s]
**SL**	**0.5137**	**0.4363 - 0.5831**	**0.2874**	**0.1933 - 0.379**
**Plates**	**0.2406**	**0.1568 - 0.3245**	0.1154	0.04257 - 0.1983
**PS**	**0.3326**	**0.2461 - 0.4154**	0.1439	0.06701 - 0.2285
**PP**	0.04434	0.003879 - 0.1027	0.06092	0.008827 - 0.1288
**DS**	0.08193	0.02771 - 0.1477	**0.2972**	**0.2032 - 0.3887**
**Eye**	**0.3617**	**0.2742 - 0.4444**	0.1005	0.03872 - 0.1728
**DF**	0.04005	0.001703 - 0.09696	0.007717	0.0003492 - 0.03266
**AF**	0.01945	0.0005656 - 0.06483	0.007589	0.0002607 - 0.03435
**Tail**	0.1593	0.0849 - 0.2392	0.07803	0.01874 - 0.1511
**BD**	**0.4124**	**0.3173 - 0.4995**	0.08696	0.02085 - 0.1672
**NLGR**	0.09442	0.03413 - 0.1653	0.03903	0.001217 - 0.1014
**GA**	0.05516	0.008992 - 0.1177	0.04339	0.005713 - 0.1058
**LGR2**	0.1571	0.08411 - 0.2361	0.05893	0.01594 - 0.1192
**LGR3**	0.106	0.04484 - 0.1774	0.08345	0.02766 - 0.1546
**LGR4**	0.08734	0.03193 - 0.1547	0.1066	0.04223 - 0.1838
**RW1**	0.06489	0.02433 - 0.1174	0.08283	0.03224 - 0.1468
**RW2**	0.2101	0.1359 - 0.287	0.1002	0.04389 - 0.168

Table S6. Genomic position of the outlier SNPs associated with the w-s branch of the population genealogy as detected by GRoSS v1.0 in three-spined stickleback ([3s]) and nine-spined stickleback ([9s]).

	Group	Position	Gene ID	Gene name	Gene description
**[3s]**	IV	19858133	ENSGACG00000018958	*pparaa*	Peroxisome proliferator-activated receptor alpha a
**[3s]**	IV	19871462	-	-	-
**[9s]**	V	6140695	ENSGACT00000003223.1	*CDHR1*	Cadherin related family member 1
**[9s]**	XII	29859509	-	-	-
